# Characterization of a novel hatching enzyme purified from starfish *Asterina pectinifera*

**DOI:** 10.1186/s40064-016-3484-7

**Published:** 2016-11-22

**Authors:** Ji Hoon Choi, Sang Moo Kim

**Affiliations:** Department of Marine Food Science and Technology, Gangneung-Wonju National University, 7 Jukheon-gil, Gangneung, 25457 Republic of Korea

**Keywords:** Deglycosylation, Hatching enzyme, Purification, Serine-zinc protease, Starfish, *Asterina pectinifera*

## Abstract

Hatching enzyme is a protease which can degrade the membrane of egg. In this study, a hatching enzyme was purified from starfish (*Asterina pectinifera*) with 6.34 fold of purification rate, 5.04 % of yield, and 73.87 U/mg of specific activity. The molecular weight of starfish hatching enzyme was 86 kDa, which was reduced to 62 kDa after removal of N-linked oligosaccharides. The optimal pH and temperature of the hatching enzyme activity were pH 7.0 and 40 °C, respectively, while those of stability were pH 8 and 20 °C. The kinetic parameters, *V*_*max*_, *K*_*m*_, K_*cat*_ and *K*_*cat*_ */K*_*m*_ values were 0.197 U/ml, 0.289 mg/ml, 112.57 s^−1^, and 389.52 ml/mg s, respectively. Zn^2+^ increased the enzyme activity by 167.28 %, while EDTA, TPCK, TGCK, leupeptin, PMSF, and TLCK decreased. In addition, Ca^2+^, Mg^2+^, and Cu^2+^ did not affect the enzyme activity. The starfish hatching enzyme activity pretreated with EDTA was recovered by Zn^2+^. Therefore, the starfish hatching enzyme was classified as a serine-zinc protease.

## Background

Hatching enzyme is a protease released from hatching gland cells in hatching embryos for digesting their protective extracellular coats (Lepage and Gache 1989; Fan and Katagiri 2001; Yasumasu et al. 1989a, b). The hatching enzyme can provide a typical model in the studies of certain cell differentiation, specific protein synthesis, and special gene expression regulation during a certain stage of early embryos at the morphological and molecular level (Fan et al. 2010). The hatching enzymes from many animal species, such as echinoderm (Lepage and Gache 1989), mammalian (Sawada et al. 1990), avians (Yasumasu et al. 2005), amphibians (Fan and Katagiri 2001; Kitamura and Katagiri 1998; Urch and Hedrick 1981), teleostean (Yasumasu et al. 1989a, b; Kudo et al. 2004; Shi et al. 2010), and insect (Young et al. 2000), have been studied since 1980s. Several marine hatching enzymes have been identified as a metalloprotease from a variety of marine species; brine shrimp *Artermia salina* (Fan et al. 2010), flounder *Paralichthys olivaceus* (Shi et al. 2010), shrimp *Penaeus chinensis* (Li et al. 2006), and sea squirt *Ciona intestinalis* (D’Aniello et al. 1997), whereas the sea urchin hatching enzyme is classified as a collagenase-like (EC 3.4.24.12) enzyme. The hatching enzymes were involved in many physiological processes such as cell migration, tissue repair, angiogenesis, inflammation, tumor invasion, and metastasis (Li and Kim 2013; Roe and Lennarz 1990).

Collagens compose about 70 % human skin, where the predominant ones are types I (80–90 %) and III (10–15 %) (Ala-Kokko et al. 1987). Hence, collagenases have been used for pharmacological purpose to treat various collagen mediated diseases such as keloid and scar, which are caused by over accumulation of collagen in tissue.

Starfish is an invertebrate belonging to the class of *Asteroidea, Phylum Echinodermata*, which produces a variety of secondary metabolites including steroids glycosides, anthraquinones, alklaoids, phospholipids, peptides, and fatty acids (Barkhouse et al. 2007; Kurihara 1999). However, starfish has been regarded as a harmful marine animal to marine ecosystem because it causes severe loss of mussel, oyster, scallop, etc. Therefore, many countries including Korea spend a lot of budget to relieve their marine ecosystem by reducing the number of starfish. In our previous studies (Li and Kim 2013, 2014a, b), a novel hatching enzyme was purified and characterized from starfish *Asterias amurensis*, which has habitat in the Ocean of East Russian. However, *Asterin apectinifera* starfish is predominant in the Ocean of Korean peninsula. Therefore, the objective of this study was to purify and characterize a hatching enzyme from starfish *A. pectinifera* for the development of a more value-added material.

## Methods

### Starfish and reagents

The adult starfish *A. pectinifera* was collected in July 2013 at Samcheok, Korea. About 500,000 live eggs were kept into 1 L of Kester artificial sea water (KASW salinity, 35.00 ‰; chlorinity, 19.00 ‰; pH 7.8) (Kester et al. 1967) and were dejellied by adjusting the pH 7.8 of KASW to 5.5 with 1 N of HCl. After 10 min, the supernatant was poured off and the precipitate was washed 3 or 4 times with the same volume of KASW. The sperms were collected out of the spermatophore artificially by pressing and were stored at 4 °C until inseminated. DEAE-sepharose fast flow and Sephacryl S-200 gels were purchased from Amersham Pharmacia Biotech (Uppsala, Sweden). Peptide-*N*-glycosidase F (PNGase F), dimethyl casein, trichloroacetic acid and tris (hydroxylmethyl) aminomethane were purchased from Sigma-Aldrich (St. Louis, MO, USA). All other chemicals and reagents that were used were of analytical grade.

### Preparation of crude hatching enzyme

A crude hatching enzyme was prepared according to the method of Lepage (1989). Briefly, approximately 100,000 eggs in 500 ml of KASW were inseminated by adding a few drops of 0.005 % sperm, stirred at 16 °C overnight, and then precipitated using 70 % ammonium sulfate at 4 °C overnight. After centrifuged at 7.728×*g* for 30 min (5810R; Eppendorf, Hamburg, Germany), the precipitate was dissolved in a 10 ml of 0.02 M Tris–HCl buffer (pH 7.4) and was then dialyzed against above buffer at 4 °C overnight. The egg membrane was prepared according to the modified method of Li (2006). About 5000 eggs were washed, stripped through 100 μm mesh, and then squeezed using a syringe needle. After washed with distilled water, the egg membrane was sonicated at 35 kHz for 10 s (MSONIC; Mirae Ultrasonic, Seoul, Korea). After centrifuged at 1.932×*g* for 15 min, the collected egg membrane was washed with distilled water completely and was resuspended in a 10 ml of 0.02 M Tris–HCl buffer (pH 7.4).

### Purification of hatching enzyme

The crude starfish extract (5 ml, 30 mg/ml) was loaded onto DEAE-Sepharose fast flow column (2.6 × 30.0 cm), and then eluted with a linear gradient of 0–1 M NaCl in 0.02 M Tris–HCl buffer (pH 7.4). The active fractions with more than 50 % maximal activity were pooled and were then dialyzed against 0.02 M Tris–HCl buffer (pH 7.4) overnight (DEAE active fraction). The DEAE active fraction was loaded onto Sephacryl S-200 gel filtration column (2.6 × 90 cm), and then eluted with 0.1 M Tris–HCl containing 0.05 M NaCl (pH 7.4). The active fractions with more than 50 % maximal activity were pooled and were dialyzed against 0.02 M Tris–HCl buffer (pH 7.4) overnight.

### Electrophoresis

The hatching enzyme was evaluated by sodium dodecyl sulfate-polyacrylamide gel electrophoresis (SDS-PAGE) with 12 % separating and 5 % stacking gels. The molecular marker (ELPIS Bioteck Co., Taejeon, Korea) ranged from 35 to 170 kDa was used to determine the molecular weight of hatching enzyme. The electrophoresized gel was stained using 0.05 % Coomassie Blue R-250 (Bio-Rad Lavoratories, Hercules, CA, USA) and was destained in a destaining solution (40 % methanol and 10 % acetic acid).

### Deglycosylation of *N*-glycans

PNGase F was used to deglycosylate the N-linked carbohydrate from the glycoproteins or glycopeptides according to the method of Sanchez et al. (2007). Twenty microlitre of the starfish hatching enzyme (200 μg) was added into 50 μl of denaturing buffer (0.5 % SDS and 1 % β-mercaptoethanol) and was then boiled for 10 min. After cooled down, 10 μl of reaction buffer (0.05 mM phosphate, pH 7.5), 5 μl of 15 % TritonX-100, and 5 μl of PNGase F (500 U/ml) were added and then incubated at 37 °C for 2 h. The reaction was stopped by heating at 100 °C for 10 min. Molecular weight of the de-*N*-glycosylated hatching enzyme was calculated based on the results of SDS-PAGE.

### Protein assay

Protein concentration of the hatching enzyme fractions was determined using Bradford method (1976). Bovine serum albumin (Sigma-Aldrich, St. Louis, MO, USA) was used as the calibration standard. The relative protein contents of chromatography fractions were estimated by measuring absorbance at 280 nm.

### Choriolytic activity

Choriolytic activity was determined according to the modified method of Yamagami (1972) using 10 mg/ml egg membrane as the substrate. Each 100 μl of the hatching enzyme and egg membrane (10 mg/ml) were mixed and incubated at 30 °C for 30 min. The reaction was stopped by adding the cold TCA (20 % w/v, 2.8 ml). After centrifuged at 3000×*g* for 30 min, the supernatant was collected. The absorbance of supernatant at 280 nm was measured using a spectrophotometer (V-300; JASCO, Seoul, Korea). One unit (U) of choriolytic activity was defined as an increase in absorbance by 0.001/min at 280 nm.

### Proteolytic activity

Proteolytic activity was determined using the determination method of choriolytic activity by substituting egg membrane with casein as the substrate. Each 100 μl of the hatching enzyme and casein (10 mg/ml) were mixed and incubated at 30 °C for 30 min. The reaction was stopped by adding the cold TCA (20 % w/v, 2.8 ml). After centrifuged at 3000×*g* for 30 min, the supernatant was collected. The absorbance of supernatant at 280 nm was measured using a spectrophotometer (V-300; JASCO). One unit (U) of proteolytic activity was defined as an increase in absorbance by 0.001/min at 280 nm.

### Effects of pH and temperature on the proteolytic activity and stability of hatching enzyme

The effect of pH profile on the proteolytic activity of hatching enzyme was determined at different ranges of pH 4.0–10.0: sodium acetic acetate buffer (pH 4.0–6.0), phosphate buffer (pH 7.0–8.0), and glycine-NaOH buffer (pH 9.0–10.0) (Li and Kim 2013). The effect of temperature on the enzyme activity was determined at different temperatures of 20–50 °C. Casein was used as the substrate. The effect of pH on the hatching enzyme stability was determined by pre-incubating enzyme over a range of pH 4.0–10.0 for 30 min. Subsequently, the enzyme mixture was adjusted to pH 7.4 using a 0.1 N NaOH or HCl. The effect of temperature on the enzyme stability was determined by pre-incubating the enzyme at 20–50 °C for 30 min. The remaining proteolytic activity for hatching enzyme activity and stability was measured under the same condition as the determination of proteolytic activity described above. The relative activity was defined as the percentage of activity determined with respect to the maximum hatching enzyme activity.

### Determination of kinetic parameters

The kinetic parameters (*K*_*m*_ and *V*_*max*_) of the purified enzyme were determined by measuring proteolytic activity at different concentrations of casein under the same condition as described above. *K*_*m*_ and *V*_*max*_ were calculated from the Lineweaver–Burk plot. The *K*_*cat*_ and *K*_*cat*_/*K*_*m*_ values were calculated based on the *K*_*m*_ and *V*_*max*_ values.

### Effect of inhibitors and metal ions on the hatching enzyme activity

The effects of various inhibitors on the proteolytic activity of hatching enzyme were determined. Each 100 μl of the hatching enzyme and casein (10 mg/ml) were mixed with inhibitors (5 mM for EDTA and EGTA, and 0.1 mM for leupeptin, TLCK, TPCK and PMSF) and incubated at 30 °C for 30 min. The reaction was stopped by adding the cold TCA (20 % w/v, 2.8 ml). After centrifuged at 3000×*g* for 30 min, the supernatant was collected. The absorbance of supernatant at 280 nm was measured using a spectrophotometer (V-300; JASCO). In addition, the purified hatching enzyme was pre-incubated at 30 °C for 30 min in the absence and the presence of bivalent cations such as Mg^2+^, Ca^2+^, Cu^2+^, and Zn^2+^. Then, the remaining proteolytic activity was measured under the same condition as described above. The relative proteolytic activity of hatching enzyme pre-incubated with no inhibitors or metal ions was used as the control.

### Recovery effect of metal ions on the EDTA-pretreated hatching enzyme

The hatching enzyme was pretreated with 10 mM of EDTA at 4 °C for 30 min. Afterwards, metal ions (Mg^2+^, Ca^2+^, Cu^2+^, and Zn^2+^) at 5 mM were added and the enzyme mixture was incubated at 4 °C for 3 h. The proteolytic activity was measured under the same condition as described above. The relative proteolytic activity of hatching enzyme pre-incubated with no metal ions was used as the control.

### Statistical analysis

Experimental results were tested in triplicates and presented as mean values ± standard error (SD).

## Results and discussion

### Purification of the starfish hatching enzyme

**Fig. 1:**
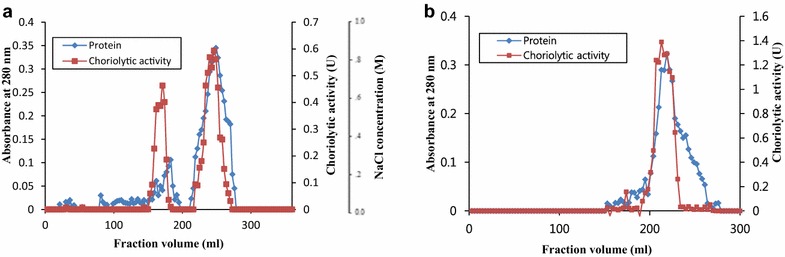
Elution profile of the starfish hatching enzyme. **a** DEAE-Ion exchange chromatography. **b** Sephachryl gel filtration chromatography

**Fig. 2:**
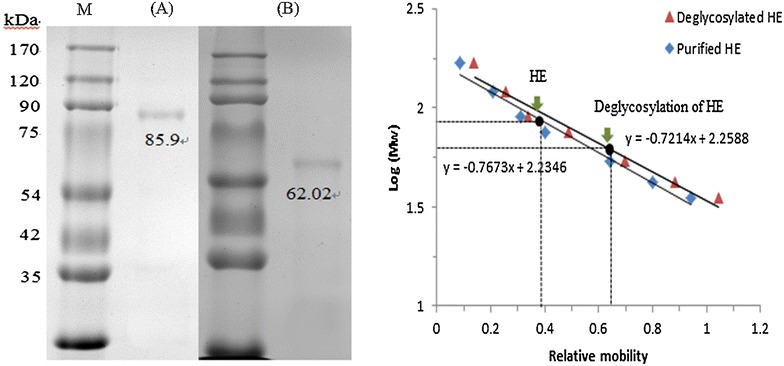
SDS-PAGE pattern of the Starfish hatching enzyme. The acrylamide concentration of the separation gel was 12 % and protein bands were stained with Coomassie* Brilliant Blue*. **A** Purified hatching enzyme from Sephacryl gel filtration.** B** N-glycan deglycosylation of the purified hatching enzyme by treatment with PNGase F. *Lane M* standard molecular weight markers

Hatching enzyme of starfish was purified using an ammonium sulfate precipitation, DEAE-Sepharose ion exchange and Sephacryl S-200 gel filtration column chromatograpy, in that order. DEAE-Sepharose ion exchange column chromatography resulted in two protein peaks with choriolytic activity (Fig. 1a). The yield, specific choriolytic activity, and purification ratio of peak I and II were 33.37 and 46.28 %, 30.22 and 23.76 U/mg, and 2.59 and 2.04 fold, respectively (Table 1). Because of higher specific choriolytic activity, the peak I was further purified using a Sephacryl S-200 column chromatography, which resulted in only one protein peak (Fig. 1b). The purification rate, yield, and specific choriolytic activity of the purified hatching enzyme were 6.34 fold, 5.04 %, and 73.87 U/mg, respectively (Table 1). The hatching enzyme with molecular weight of 86 kDa was homogeneous on the SDS-PAGE (Fig. 2a). The specific choriolytic activity (73.87 U/mg) of the starfish hatching enzyme in this study was lower than 400.00 U/mg of brine shrimp (Fan et al. 2010) and 449.62 U/mg of starfish *A. amurensis* (Li and Kim 2013). The purification rate and yield of starfish hatching enzyme (6.34 fold and 5.04 %) in this study were also lower than those of starfish *A. amurensis* (7.42 fold and 14.28 %) (Li and Kim 2013), shrimp (48.05 fold and 44.29 %) (Li et al. 2006), sea urchin (201 fold and 53 %) (Roe and Lennarz 1990), and sea squirt (67.8 fold and 29.4 %) (D’Aniello et al. 1997). These differences might be due to different species, preparations, and purification methods. The molecular weight of the starfish hatching enzyme in this study was 86 kDa, which was a smaller than 110.9 kDa of *A. amurensis* (Li and Kim 2013), but a little higher than 73.3 kDa of brine shrimp (Fan et al. 2010). However, it was much higher than those of the hatching enzyme from shrimp (43 kDa) (Li et al. 2006), sea urchin (37, 44, 51 kDa) (Lepage and Gache 1989; Nomura et al. 1991; Takeuchi et al. 1979), frog (40, 56 kDa) (Fan and Katagiri 2001; Kitamura and Katagiri 1998), sea squirt (34 kDa) (D’Aniello et al. 1997), flounder (34.8 kDa) (Shi et al. 2010), *Fundulus heteroclitus* (15–40 kDa) (DiMichele et al. 1981), *Oryzias latipes* (LCE 25.5 kDa; HCE 24 kDa) (Yasumasu et al. 1989a, b), and *Salmo gairdneri* (10 kDa) (Hagenmaier 1974). The PNGase F was used to release the asparagine-linked (N-linked) oligosaccharides from the hatching enzyme protein. After PNGase F treatment, the band of hatching enzyme protein with 86 kDa was shifted to 62 kDa (Fig. 2b). Therefore, the 24 kDa of N-linked oligosaccharides was removed from the hatching enzyme protein. The molecular weight of the starfish hatching enzyme, 86 kDa, was quietly different from those of other animals (shrimp, sea urchin, frog, sea squirt, flounder, mummichog, medaka, and rainbow trout) including 110.9 kDa of starfish *Asterias amurensis*. In addition, Tbrain or T-box brain protein 1 is a transcription factor protein important in vertebrate embryo development. It is encoded by the TBR1 gene which is involved in the mesoderm formation of vertebrate embryos. Mammalian T-brain is expressed in the developing central nervous system. Hinman et al. (2007) reported the results of gene analysis of sea stars and sea urchins as follows; it has been conserved for 500 million years since sea stars and sea urchins last shared a common ancestor. Amid this high level of conservation, one significant regulatory change was elucidated. Tbrain was required for correct otxβ1/2 expression in the sea star, but not in the sea urchin. In sea urchin, Tbrain was not co-expressed with otxβ1/2 and instead had an essential role in specification of the embryonic skeleton. Tbrain in these echinoderms was thus a perfect example of an orthologous gene co-opted for entirely different developmental processes. According to above explanations, the starfish hatching enzyme might be ortholog.

**Table 1:** Purification of hatching enzyme from starfish *Asterinapectinifera*

Purification step	Total protein (mg)	Total choriolyticactivity (U)	Specific**c**horiolytic activity (U/mg)	Yield (%)	Purification (fold)
Hatching crude	94.21	1098.50	11.66	100	1
Ion exchange					2.59
Peak I	12.13	366.60	30.22	33.37	
Peak II	21.4	508.40	23.76	46.28	2.04
Gel filtration					6.34
Peak I	0.75	55.40	73.87	5.04

### Effect of pH and temperature on the hatching enzyme activity and stability

**Fig. 3:**
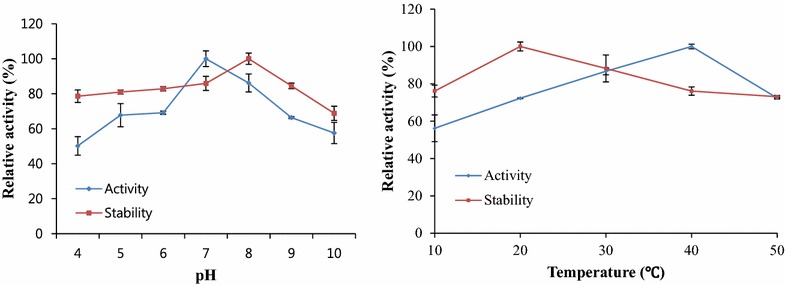
Effects of pH and temperature on the proteolytic activity and stability of hatching enzyme

The starfish hatching enzyme exhibited a higher activity in the range of pH 6.0–9.0 and maximum activity at pH 7.0 (Fig. 3). This enzyme was stable at pH 6.0–9.0 and had the maximum stability at pH 8.0 (Fig. 3). The maximum activity pH (7.0) of the starfish hatching enzyme in this study was the same as pH 7.0 of brine shrimp and lower than those of the hatching enzyme of sea urchin (pH 8.0) (Roe and Lennarz 1990; Li and Kim 2014), *A. amurensis* (pH 8.0) (Li and Kim 2013), *O. latipes* (LCE pH 8.6; HCE pH 8.0, 8.7) (Yasumasu et al. 1989a, b), *S. gairdneri* (pH 8.0–8.5) (Hagenmaier 1974), sea squirt (pH 8.5) (D’Aniello et al. 1997), quail (pH 9.0) (Iwasawa et al. 2009), but higher than pH 6.0 of the shrimp hatching enzyme (Li et al. 2006). The optimal activity temperature of the starfish hatching enzyme was 40 °C (Fig. 3), whereas its maximum stability temperature was 20 °C (Fig. 3). The optimal activity temperature of the starfish hatching enzyme was the same as 40 °C of the sea urchin (Nomura et al. 1991), brine shrimp (Fan et al. 2010), and shrimp (Li et al. 2006), but higher than 30 °C of frog (Roe and Lennarz 1990; Kester et al. 1967), *O. latipes* (Yasumasu et al. 1989a, b), and *A. amurensis* (Li and Kim 2013). These stable pH and temperature of the starfish hatching enzyme are important to skincare because the acidic pH (4.4–5.6) and the imbalance change in skin permit for normal exfoliation of surface dead cells well (Natalia and Varinia 2010). Furthermore, in the early state of injury or wound healing, the considerable fibrinogen from the liver is deposited as fibrin or fibronectin on the gap of the damage part (Brown et al. 1993). Meanwhile, the dermal fibroblasts begin to cluster to this fibrin matrix, over-accumulate collagen and then built the skin contraction as collagen-like tissue (Clark 1993). Hence, the over-accumulation of collagen is responsible for the unsmooth skin of scar or keloid. Li and Kim (2014) reported that the *A. ammurensis* starfish hatching enzyme had comparable ability to collagenase and α-chymotrypsin, which degraded collagen and fibrinogen efficiently. In addition, the *A. ammurensis* starfish hatching enzyme had the potential application to remove the matrix composition in scar or keloid tissue. It is generally known that the temperature and pH of human skin are 28–32 °C and pH 7.0, respectively (Plasencia et al. 2007). Therefore, the *A. pectinifera* starfish hatching enzyme which was very stable at pH 7.0 and 20–30 °C might have a potential for the development of a skin care product.

### Effects of chelators, inhibitors, and metal ions on the enzyme activity

**Fig. 4:**
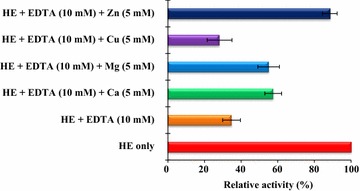
Recovery effect of metal ions on the EDTA pretreated starfish hatching enzyme

The effects of chelators, inhibitors, and metal ions on the enzyme activity are shown in Table 2. EDTA and EGTA inhibited significantly the proteolytic activity of hatching enzyme by more than 50 % (Table 2), which was similar to the results of the frog (Fan and Katagiri 2001), flounder (Shi et al. 2010), sea squirt (D’Aniello et al. 1997), *A. amurensis* (Li and Kim 2013), and sea urchin (Roe and Lennarz 1990). The proteolytic activity of hatching enzyme was strongly activated by 167.28 % at 5 mM of Zn^2+^ (Table 2). Zn^2+^ also recovered the denatured hatching enzyme activity more greatly than other ion metals (Fig. 4), which was similar to the hatching enzymes of the brine shrimp (Fan et al. 2010), sea squirt (D’Aniello et al. 1997), *A. amurensis* (Li and Kim 2013) and shrimp (Li et al. 2006). Based on the inhibitory activity of EDTA and EGTA, the starfish hatching enzyme in this study was characterized as metalloprotease, which was similar to the hatching enzymes of sea squirt (D’Aniello et al. 1997) and sea urchin (Roe and Lennarz 1990). TLCK and TPCK are known to inhibit trypsin and chymotrypsin through the alkylation of a histidine residue at active sites, whereas PMSF and leupeptin inhibit them by sulfonylating the hydroxyl group of the serine residue at the active site, respectively (Ikegami et al. 1994). The starfish hatching enzyme was sensitive to EDTA and several metal ions (Table 2). Zn^2+^ recovered the proteolytic activity of starfish hatching enzyme pretreated with EDTA. Therefore, it was indicated that starfish hatching enzyme might be also a kind of Zn^2+^-protease, which was similar to the results of hatching enzymes from frog (Fan and Katagiri 2001; Kitamura and Katagiri 1998), *O. latipes* (Yasumasu et al. 1989a, b), brine shrimp (Fan et al. 2010), flounder (Shi et al. 2010), shrimp (Li et al. 2006), sea squirt (D’Aniello et al. 1997), *A. amurensis* (Li and Kim 2013), *F. heteroclitus* (DiMichele et al. 1981), sea urchin (Yasumasu et al. 1989), and pike (Schoot and Denuce 1981). Based on these results, the *A. pectinifera* starfish hatching enzyme was classified as a serine-zinc protease.

**Table 2:** Effect of metal ions and inhibitors on the proteolyticactivity of hatching enzyme

Inhibitors or metal ions	Concentration (mM)	Relative activity (%)
EDTA	5	38.15 ± 9.86
EGTA	5	42.31 ± 8.41
Cu^2+^	10	75.38 ± 7.01
Mg^2+^	10	71.22 ± 4.65
Zn^2+^	10	167.28 ± 12.69
Ca^2+^	10	86.47 ± 2.50
Leupeptin	0.1	56.29 ± 2.57
PMSF	0.1	56.20 ± 4.15
TLCK	0.1	56.72 ± 2.34
TPCK	0.1	40.47 ± 8.40

### Kinetic parameters

**Fig. 5:**
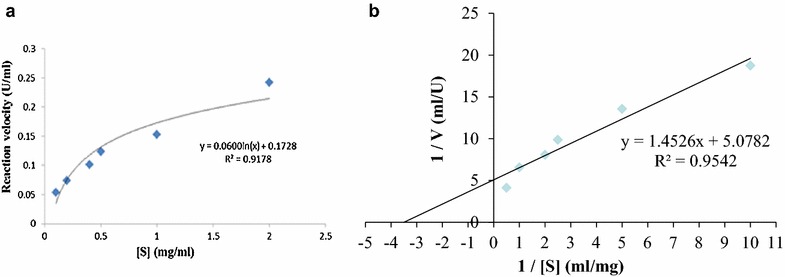
Michaelis–Menten kinetic curve **a** and Lineweaver-Bulk plots **b** of the starfish hatching enzyme

The kinetic parameters (*K*_*m*_, *V*_*max*_) of the purified hatching enzyme were determined by measuring proteolytic activity at different concentrations of casein (Fig. 5). The *K*_*m*_, *V*_*max*_, *K*_*cat*_, and *K*_*cat*_ */K*_*m*_ values of the starfish hatching enzyme were 0.289 mg/ml, 0.197 U/ml, 112.57 s^−1^, and 389.52 ml/mg s, respectively. *K*_*m*_ value (0.289 mg/ml) of the starfish hatching enzyme on casein was lower than 8.20 mg/ml of brine shrimp (Fan et al. 2010), 7.47 mg/ml of shrimp (Li et al. 2006), 4.28 mg/ml of flounder (Shi et al. 2010), and 0.31 mg/ml of starfish *A. amurensis* (Li and Kim 2013), whereas higher than 0.2 mg/ml of frog (Fan and Katagiri 2001) and 0.12 mg/ml of sea urchin (Roe and Lennarz 1990). The diversities of the *K*_*m*_ value may be correlated with the difference in species, survival environments, enzyme structures, and ion concentrations as well. The less *K*_*m*_ value means the higher affinity of enzyme to substrate (Ranaldi et al. 1999). Therefore, it was thought that the starfish hatching enzyme might be efficient for the degradation of collagen.

## Conclusions

A novel hatching enzyme with 86 kDa of molecular weight was purified from starfish (*A. pectinifera*). De-*N*-glycosylation of the enzyme leads to a loss of 24 kDa as observed by the migration behavior in SDS-PAGE. The purification rate and yield of starfish hatching enzyme were 6.34 fold and 5.04 %, respectively. The optimal pH and temperature of hatching enzyme activity were 7.0 and 40 °C, respectively, while those of stability were pH 7.0 and 20 °C. The starfish hatching enzyme was classified was a serine-zinc protease. Therefore, the *A. pectinifera* hatching enzyme might be utilized as a cosmeceutical because its optimum pH and temperature stability were similar to those of human skin.
